# Generating microglia from human pluripotent stem cells: novel in vitro models for the study of neurodegeneration

**DOI:** 10.1186/s13024-019-0347-z

**Published:** 2019-12-19

**Authors:** Anna M. Speicher, Heinz Wiendl, Sven G. Meuth, Matthias Pawlowski

**Affiliations:** Department of Neurology with Institute of Translational Neurology, University Hospital Münster, Albert-Schweitzer-Campus 1, Building A1, 48149 Münster, Germany

**Keywords:** Human microglia, Human pluripotent stem cells, Embryonic development, Differentiation, Human in vitro models, Neuroinflammation, Neurodegeneration

## Abstract

Microglia play an essential role for central nervous system (CNS) development and homeostasis and have been implicated in the onset, progression, and clearance of numerous diseases affecting the CNS. Previous in vitro research on human microglia was restricted to post-mortem brain tissue-derived microglia, with limited availability and lack of scalability. Recently, the first protocols for the generation of microglia from human pluripotent stem cells have become available, thus enabling the implementation of powerful platforms for disease modeling, drug testing, and studies on cell transplantation. Here we give a detailed and comprehensive overview of the protocols available for generating microglia from human pluripotent stem cells, highlighting the advantages, drawbacks, and operability and placing them into the context of current knowledge of human embryonic development. We review novel insights into microglia biology and the role of microglia in neurological diseases as drawn from the new methods and provide an outlook for future lines of research involving human pluripotent stem cell-derived microglia.

## Background

Microglia are the resident immune cells of the central nervous system (CNS). They are derived from yolk sac macrophages that arise during the first wave of primitive hematopoiesis and populate the developing CNS via the bloodstream once embryonic circulation is established [1, 2]. Once the blood-brain barrier is formed, the influx of yolk sac macrophages into the CNS ceases [3], and the early microglia population is self-maintained throughout life by slow continuous turnover [4]. Microglia play a crucial role during healthy CNS development and support CNS homeostasis throughout adult life [5]. Moreover, microglia are involved in the initiation, progression, and clearance of diseases affecting the CNS [6, 7]. Recently, microglia have entered center stage in neurodegenerative disease research even though their exact function during the disease course remains unclear [8, 9]. This advancement was fueled by the discovery that many of the genes identified as risk factors for Alzheimer’s disease and other neurodegenerative diseases are preferentially expressed by microglia and that microglia display disease-stage dependent capacities to clear pathological aggregates of misfolded proteins and neuronal debris [10, 11]. Although the use of animal models and primary murine microglia in vitro cultures have greatly expanded our understanding of microglia, species-specific variations often prevent the translation of microglia-related research findings from rodent models to humans, especially in the context of age-related neurodegenerative diseases [12–14]. Therefore, faithful human microglia models are required to elucidate the cellular and molecular phenotypes and pathways related to homeostatic, neuroprotective, and neurotoxic microglia. Until recently, the isolation of microglia from human post-mortem brain tissue or diseased neurosurgical specimen represented the only option for in vitro studies of human microglia [15–18]. The capture of human embryonic stem cells (hESCs) in vitro [19] and the generation of human-induced pluripotent stem cells (hiPSCs) [20], collectively referred to as human pluripotent stem cells (hPSCs), has enabled researchers to develop protocols for generating all cell types of the organism from an indefinite and renewable source. But while protocols for many cell types, including all principle neuroectoderm-derived neural cell types, were promptly established and intensively studied and refined, the production of microglia initially remained elusive. Only by the end of 2016, the first dedicated differentiation protocol for the generation of human microglia-like cells was published. This impediment may be attributed to the peculiar embryonic origin of microglia that is distinct from all other tissue macrophages and was discovered only recently [1, 2]. Understanding microglial ontogeny is an indispensable prerequisite for their generation from hPSCs employing classical differentiation. Since late 2016, several protocols for generating microglia-like cells (MGLs) from hPSCs have been published [21]. Most protocols aim to mimic the sequel of embryonic development by timed, stage-specific exposure of hPSCs and their progeny to individual growth factors or small molecules. For the first time, this approach provides a tool for the scalable production of human MGLs. However, the published protocols differ substantially concerning the length of the required culture periods, the need for mechanical manipulation or fluorescence-activated cell sorting (FACS) steps to enrich for intermediate progenitor populations, and the functional and molecular phenotypes of the MGLs produced. Moreover, in some protocols, hPSCs are probably differentiated into monocytes. These are subsequently exposed to extracellular cues, resulting in macrophages with microglia-like properties or their transdifferentiation into MGLs. The use of monocyte-derived cells as a human microglia model may be contentious, though, because of the distinct ontogeny of microglia and peripheral tissue macrophages. Here, we provide a detailed review of currently available protocols for the generation of MGLs from hPSCs. We highlight distinctive features of the different protocols and compare the phenotypes of the resulting MGL products. Finally, we present a comprehensive overview of published translational applications of MGLs and outline future directions of microglia research using MGLs.

## Main text

### Protocol 1 – Muffat et al

Muffat et al. reported the first dedicated protocol for the generation of MGLs from hPSCs [21]. Interestingly, their approach deviates substantially from all other subsequent protocols. The starting point is the generation of embryoid bodies (EBs) by treating hPSC colonies with collagenase IV, followed by mild trituration and plating of undefined clumps on low-adhesion tissue culture (TC) plates in the final differentiation medium. The chemically defined medium used from day 0 onwards is not changed throughout the protocol and contains the two growth factors colony-stimulating factor 1 (CSF1) and interleukin (IL-)34. Thus, stage-specific extracellular cues directing the cells through the sequel of embryonic intermediates are not provided. Nevertheless, the authors provide convincing evidence that the cells pass through key developmental microglia-lineage stages. First, between day 14 and 44, several EBs form cystic structures. Upon plating those structures onto poly-D-lysine (PDL)-coated TC dishes, they form flat cell lawns reminiscent of endothelial cells and express the transcription factor (TF) PU.1 (*SPI1*) and the cell surface markers CD41 (integrin subunit *ITGA2B*), CD117 (*KIT*), CD144 (cadherin 5, *CDH5*), and CD235a (glycophorin A, *GYPA*). The authors refer to the cystic structures as “yolk sac EBs” resembling hemogenic epithelium. From the floating yolk sac EBs, single cells delaminate into the TC medium, which can be collected and plated onto Primaria™ TC plates, providing selective adhesive conditions for myeloid progenitors. Within the first 2 weeks of plating onto Primaria™ plates, these cells resemble MGL precursors. They are round, vacuolated cells with a compact nucleus, filopodia, and membrane ruffles, expressing the TF PU.1, the intracellular calcium-binding protein IBA1 (*AIF1*), and the surface markers CD11b and CD45 (*PTPRC*). They are highly proliferative and capable of latex bead phagocytosis. After an additional month of differentiation in monoculture, MGL precursors differentiate into MGLs and start expressing the purinergic receptor P2Y12 (*P2RY12*) and the transmembrane protein TMEM119 in addition to IBA1 and CD45. At this later stage of microglial development, the proliferation rate is drastically reduced, nevertheless, MGLs could be maintained in culture for several months. Mature microglia that were generated using this method were compared to freshly isolated fetal human microglia that were cultivated in the same medium for 1 week. The transcriptome analysis revealed that both cell types express the same set of microglial signature genes. Upon culturing MGLs in neural precursor cell (NPC)-conditioned media for 2 weeks, the transcriptome of MGLs shifted closer towards primary microglia ex vivo. Finally, 3D aggregates were formed by placing differentiating NPC cultures containing neurons, macroglia, and MGLs into low attachment TC dishes: integrated MGLs exhibited a more ramified morphology compared to 2D cultures, and their arborization was rapidly extended and retracted during tiling of the aggregate. Following the generation of a focal laser-induced lesion within the 3D aggregate, integrated MGLs in the vicinity of the lesion reacted within minutes by extending a single long process towards the lesion site and migrating to surround the damaged area [21].

### Protocol 2 – Pandya et al

Pandya et al. developed a two-step protocol for the generation of MGLs from hPSCs [22]. First, adherent hPSC colonies are passaged as single cells and maintained in pluripotency media at low oxygen conditions for 2 days. Subsequently, cells are differentiated in commercial differentiation medium (STEMdiff APEL) under hypoxic conditions for 15 days. During the first 4 days, the medium is supplemented with the growth factors bone morphogenetic protein 4 (BMP4), stem cell factor (SCF, *KITLG*), vascular endothelial growth factor A (VEGF-A), and activin A (a homodimer of two inhibin βA chains, *INHBA*). For the remaining culture period, the latter two are replaced by IL-3, IL-6, Flt3 ligand (*Flt3LG*), and CSF3. By day 15 of the protocol, a small fraction of cells is floating in the culture supernatant. These cells express CD45 and the hematopoietic progenitor surface markers CD34 and CD43. They are transferred onto a layer of “human astrocytes”, although the source and properties of these astrocytes are not reported. During the subsequent coculture period, the medium is supplemented with undefined FBS 10% and the growth factors IL-3, CSF1, and CSF2. Following 2 weeks of coculture, the cells have downregulated their expression of CD34 and CD43 and have started to express CD11b, CD39 (*ENTPD1*), CX3CR1, IBA1, and HLA-DR in addition to CD45. A subpopulation also expresses low levels of TREM2. Gene expression analysis using microarrays showed similarities of MGLs to human fetal microglia but also dendritic cells and macrophages. Genes related to the “microglia signature” (e.g., *P2RY12*, *GPR34*, *MERTK*, *C1QA*, *PROS1*, and *GAS6*) showed higher expression levels in MGLs than in dendritic cells, macrophages, and hPSCs. Functional assays demonstrated that MGLs are capable of phagocytosis, reactive oxygen species (ROS)-production, and secretion of tumor necrosis factor alpha (TNFα) upon stimulation with LPS. With an output of 0.8–3 times the number of MGLs compared to the starting material, the yield is rather low, and extracted from the microglia–astrocyte coculture, the purity of MGLs is as low as 9% [22].

### Protocol 3 – Abud et al

Abud et al. start by passaging hiPSC colonies as single cells [23]. On the following day, adherent hiPSCs are differentiated in chemically defined media by transient, stage-specific exposure to growth factors mimicking the extracellular signals during embryonic development. During the first 2 days of differentiation, the medium is supplemented with fibroblast growth factor 2 (FGF2), BMP4, and activin A. From day 2 to day 4, the latter two are withdrawn and replaced by VEGF-A. Subsequently, thrombopoietin (TPO, *THPO*), SCF, IL-3, and IL-6 are added alongside FGF2 and VEGF-A. By day 10 of the protocol, a small fraction of cells is floating in the culture supernatant. These cells represent hematopoietic progenitors that express the cell surface markers CD41, CD43, and CD235a. At this stage, the hematopoietic progenitors are enriched by FACS for CD43^+^ cells, which are transferred onto fresh Matrigel-coated TC dishes and cultivated for another 28 days in chemically defined microglia differentiation medium containing CSF1, IL-34, and TGF-β. For the final 3 days of maturation, the medium is further supplemented with CD200 and CX3CL1. The resulting MGLs at day 38 of differentiation express the TF PU.1 and the cell surface markers CD11b, CD45, CX3CR1, TREM2, TGFβR1, P2Y12, MERTK, PROS1, and ITGB5. Principal component analysis of RNA-seq data showed clustering of MGLs with both fetal and adult primary microglia but also proximity to hematopoietic progenitors at day 10 of the differentiation protocol. Undifferentiated hiPSCs, peripheral blood dendritic cells, and CD14^+^/CD16^+^ non-classical monocytes clustered away from MGLs. Both the addition of CD200 and CX3CL1 and the coculture of MGLs with matured primary rat hippocampal neurons for the last 3 days of microglia maturation led to differential gene expression indicating a transcriptional phenotype that aligns more closely with primary microglia. Functional assays demonstrated that MGLs are capable of phagocytosis, responsive to ADP-signaling by producing P2Y12-dependent intracellular Ca^2+^ transients, and secrete typical cytokines following stimulation with either LPS, IL-1β, or IFN-γ. Moreover, MGLs are capable of CD11b-dependent, MERTK-independent phagocytosis of human synaptosomes – a typical microglial pathway of synaptic pruning. Likewise, MGLs internalize fibrillar Aβ (fAβ) and brain-derived tau oligomers (BDTO), and quantitative real-time PCR (qPCR) of MGLs stimulated with fAβ or BDTO confirmed an increased expression of several genes implicated in Aβ clearance or tau-mediated toxicity. MGLs cocultured with brain organoids (BORGs) invade the 3D CNS environment, extend ramified processes, and respond to needle piercing of the BORGs by migrating towards the site of injury and adopting an amoeboid-like appearance. Similarly, xenotransplantation of MGLs into humanized immunodeficient MITRG mice (Rag2^−/−^Il2rg^−/−^ mice in which genes encoding human CSF1, CSF2, IL-3, and TPO were knocked-in the respective mouse orthologues) demonstrated engraftment and long-term survival of highly branched, IBA1^+^/P2Y12^+^/TMEM119^+^ human cells within the mouse brain. The yield of MGLs is as high as 30–40 times the starting cell number and the purity reaches almost 100%, although the protocol requires FACS to enrich for hematopoietic progenitors [23]. The same lab recently published a follow-up protocol, in which the low oxygen culture conditions from day 0 to 4 and the FACS step to enrich for hematopoietic progenitors are omitted, and the commercial Stem Cell Technologies STEMdiff™ Hematopoietic Kit is used for generating hematogenic progenitors. From day 10 to 38 of differentiation, TGF-β is replaced by the small molecule IDE1 (inducer of definitive endoderm 1), which induces TGF-β signaling by phosphorylating Smad2/3 and increasing NODAL expression [24]. At day 10 of the protocol, the culture supernatant is collected, and floating cells are transferred to a new TC dish without enriching specific cell populations. This measure leads to a 60-fold higher yield of hematopoietic progenitors, while purity is reduced to 90% and CD41 and CD43 are expressed at lower levels. Moreover, expression of CD235a was hardly detectable in hematopoietic progenitors (2%; compared to 10% in the previous version of the protocol) [24]. The reduced expression of CD235a may be a result of the maturity of the cells, as noted by principal component analysis. With regard to the final MGL product, transcriptome analysis by RNA-seq demonstrated a high degree of similarity between both MGL products, although slightly different gene expression patterns were evident and should be considered when planning downstream applications. Taken together, the changes in the updated protocol enable easier handling and provide a more cost-effective alternative to the original protocol, however, the phenotype of the intermediate hematopoietic progenitor population is altered, and their purity is reduced, while total culture durations remain unchanged.

### Protocol 4 - Douvaras et al

Douvaras et al. start by passaging hPSC colonies as single cells [25]. After 3 days, once new individual colonies become apparent, differentiation is induced in chemically defined, commercial media (mTeSR/StemPro) by transient, stage-specific exposure to growth factors aiming to mimic extracellular signaling during embryonic development. During the first 4 days of differentiation, the medium is supplemented with BMP4. On day 4, BMP4 is withdrawn and replaced by FGF2, VEGF-A, and SCF. The authors describe adherent endothelial cells to be present on day 6. Flow cytometry revealed a small shift in the expression of CD235a and CD309, suggesting co-expression of both markers by up to 6% of the total cell population. From day 6 onwards, the medium is supplemented with TPO, Flt3 ligand, IL-3, and CSF1 alongside SCF. From day 14 onwards, the medium contains Flt3 ligand, CSF1, and CSF2. Starting from day 25, MGL precursors, defined by co-expression of CD14, CD45, and CX3CR1, detach from the adherent hematopoietic progenitors and may be harvested and enriched by FACS or magnetic bead separation (MACS) weekly for up to 1 month. Selected cells are transferred onto Thermanox™ coverslips and cultivated for another 20 days in chemically defined microglia differentiation medium containing CSF2 and IL-34. The resulting MGLs express IBA1, CD11b, CD11c, CX3CR1, P2Y12, and TMEM119. Hierarchical cluster analysis of whole-transcriptome RNA-seq data, based on either all or only selected microglial signature genes, confirmed clustering of MGLs and primary microglia and their segregation from different types of macrophages. Functional assays demonstrated that MGLs are capable of phagocytosis, responsive to ADP-signaling by producing P2Y12-dependent intracellular Ca^2+^ transients, and secrete several cytokines, although MGLs were not stimulated. The protocol has a rather low efficiency: only 68% of the cells of the final cell population express CD14 that is not uniquely expressed by microglia but also by other cell types of the myeloid lineage [25].

### Protocol 5 – Haenseler et al

James and co-workers had previously reported protocols for the generation of monocytes and macrophages from hPSCs [26, 27]. Only later, it was discovered that these presumptive monocytes/macrophages develop independently of the TF MYB and rely on the expression of RUNX1 and PU.1, suggesting that these myeloid cells rather epitomize primitive yolk sac macrophages [28]. In a follow-up study, Haenseler et al. directed the fate of these yolk sac macrophage-like cells towards MGLs [29]. The differentiation protocol starts with EB formation. During the first 4 days, EBs are cultivated in commercial mTeSR1 medium supplemented with BMP4, VEGF-A, and SCF. On day 4, EBs are transferred into larger TC dishes and cultivated in chemically defined, commercial X-VIVO medium supplemented with IL-3 and CSF1. Most EBs put out surrounding adherent stromal cells and develop cystic, yolk-sac-like structures similar to the ones described by Muffat et al. [21]. After 3–4 weeks, MGL precursors emerge in the supernatant as large, round cells with filopodia and membrane ruffles. These cells may be harvested and transferred onto uncoated plastic TC dishes or hPSC-derived neuronal cells. Within 2 weeks after transfer, MGLs develop in chemically defined medium supplemented with CSF2 and IL-34. The resulting MGLs display secondary branches and express CD11b, CD11c, IBA1, CX3CR1, and P2Y12. Faint positive staining was also reported for TMEM119. In contrast, HLA-DR expression was not detected. Principal component analysis using microarray data confirmed clustering of MGLs in mono- or coculture with fetal microglia and hPSCs-derived macrophages but segregation from human peripheral blood-derived monocytes and hPSC-derived macrophage precursors. Functional assays demonstrated that MGLs are capable of phagocytosis and secrete several cytokines upon stimulation with LPS and IFN-γ. Live cell imaging of fluorescently-labeled MGLs in coculture with hPSC-derived neurons displayed continually moving MGLs within the neuronal culture, thereby roughly maintaining cell-specific territories, and dynamic remodeling of primary and secondary cellular branches [29].

### Protocol 6 – Takata et al

Takata et al. start by passaging adherent hPSC colonies as clumps [30]. On the following day, adherent hPSCs are differentiated in chemically defined, commercial (StemPro) differentiation medium by transient, stage-specific exposure to growth factors mimicking the extracellular signals during embryonic development. From day 0 to day 8 of differentiation, the cells are cultivated in hypoxic conditions (O_2_ 5%), while the remaining protocol is performed in normoxic conditions. During the first 2 days of differentiation, the medium is supplemented with VEGF-A, BMP4, and CHIR99021 (a selective GSK-3 inhibitor that activates canonical WNT-signaling). From day 2 to day 4, CHIR99021 is replaced by FGF2, and from day 4 to day 6, BMP4 is withdrawn. Subsequently, SCF, IL-3, IL-6, and DKK1 are added alongside FGF2 and VEGF. From day 12 to day 16, the medium is supplemented with SCF, IL-3, and IL-6. The commercial StemPro base medium is replaced between day 16 and day 26 by a chemically defined medium supplemented with CSF1. By day 26, a fraction of cells is floating in the culture supernatant. These cells are largely positive for the surface markers CD11b, CD14, CD45, CD163, and CX3CR1. While the media composition follows some rationales of embryonic development, there is no proof of developmental intermediates from the posterior primitive streak, extra-embryonic mesoderm, hemangioblast, or primitive hematopoiesis. After 26 days of differentiation, MGL precursors are collected from the supernatant and transferred onto hPSC-derived neurons. Over the course of another 3 weeks, the primitive macrophages develop a more ramified morphology and start to express IBA1. MGL precursors and MGLs were shown to phagocyte latex beads and Aβ peptides and release typical pro-inflammatory cytokines upon stimulation with LPS [30].

### Additional MGL protocols

Amos et al. and Xu et al. reported further protocols for the generation of MGLs [31, 32]. Initially, Amos et al. generate EBs that are exposed to chemically undefined media containing BMP4 and FBS 10% [31]. EBs are transferred onto poly-L-ornithine/fibronectin-coated TC plates on day 7 and cultivated for another 33 days in commercial microglia medium containing FBS alongside CSF1, CSF2, IL-34, and TGFβ-1. From day 23 onwards, EBs are mechanically passaged every 4–6 days. Similar to the protocol by Muffat et al., two types of EBs emerge: cystic EBs and dense, non-cystic EBs. Upon plating the cystic EBs onto coated TC dishes, a “skirt” of MGL precursors detaches from the cystic structures and migrates away. However, this phenomenon was observed only in one-third of cystic EBs. Flow cytometry of MGLs demonstrated expression of CX3CR1 in 4% and TREM2 in 20% of cells, while TMEM119 expression was not detected. MGLs phagocyte Aβ and upregulate *IL1B*, *CCL2*, and *TNFAIP3* expression upon stimulation with LPS, as demonstrated by qPCR. Furthermore, the qPCR analysis confirmed the upregulation of *CSF1R*, *CX3CR1*, *P2RY12*, and *HEXB* compared to peripheral blood-derived monocytes and hPSCs, while transcriptome analysis and further functional assays were not performed [31]. Xu et al. present a protocol that is related to the Abud et al. protocol [32]. The authors use a commercial kit (STEMdiff™ Hematopoietic Kit) to generate hematopoietic progenitors from hPSCs. On day 12, hematopoietic progenitors are enriched by FACS according to CD43 expression and further differentiated in chemically undefined, commercial, serum-containing medium (Microglia Medium, ScienCell Research) supplemented with insulin-like growth factor 1 (IGF-1), CSF1, CSF2, IL-34, and TGF-β. The authors report that the resulting MGLs co-express IBA1 and TMEM119 following a total culture period of 37 days, although morphologically, only a few short cell processes are detected. Functional assays demonstrated that MGLs are capable of phagocytosis and TNF-α release upon stimulation with LPS, but transcriptome analysis and further characterization were not performed [32].

**Fig. 1:**
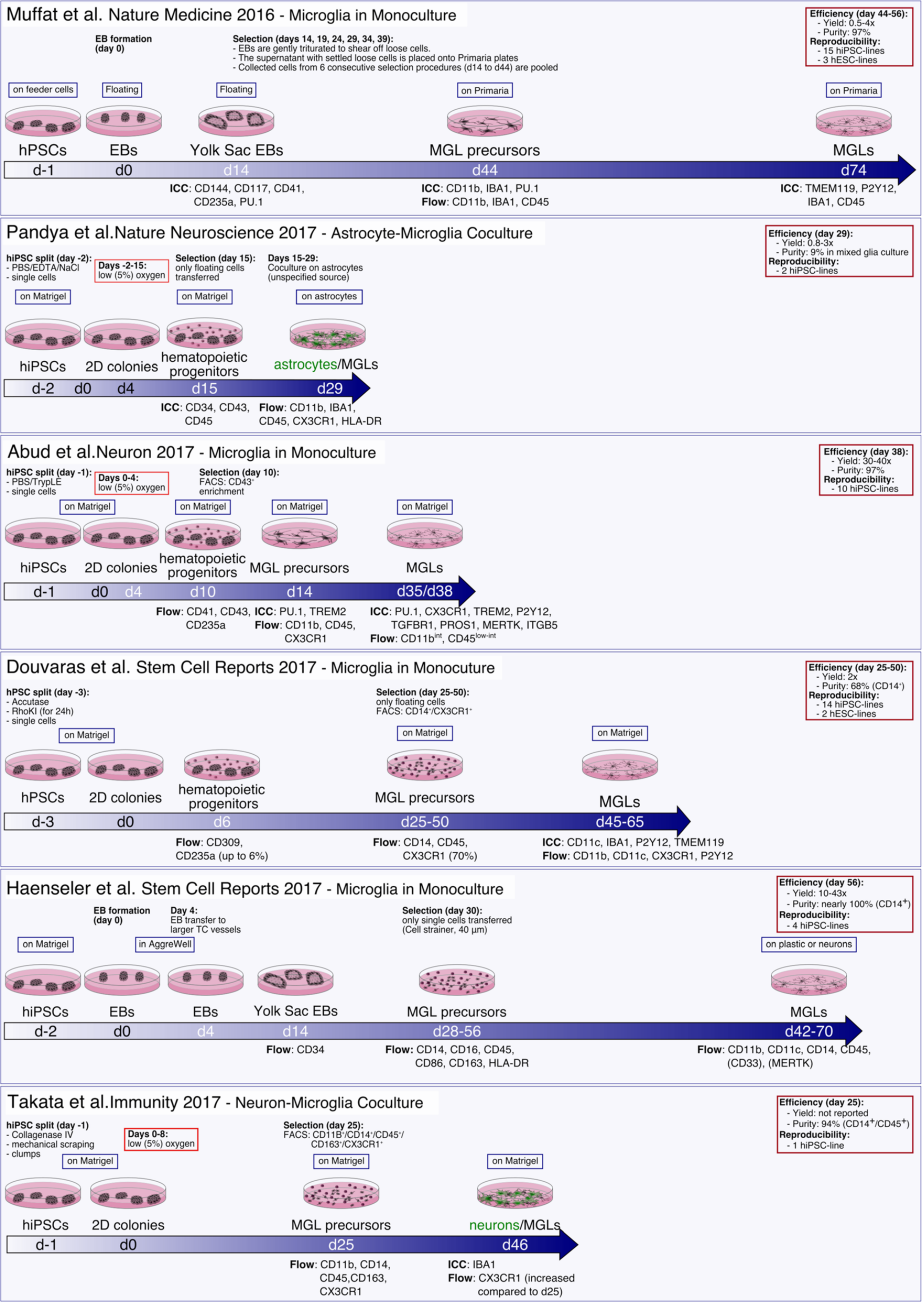
Schematic overview of protocols for generating microglia-like cells from human pluripotent stem cells

In Fig. 1, we summarize the first six protocols described here for generating MGLs. Detailed stage-specific media compositions are reported in Table 1. Marker protein and gene expression analyses, and metabolic and functional studies are summarized in Table 2. Protocols by Amos et al. and Xu et al. are not included as they involve chemically undefined culture conditions.

**Table 1:** Detailed summary of media compositions used for the differentiation of hPSCs into microglia-like cells

	hPSCs	Differentiation to Hematopoietic Progenitors					Differentiation to Microglia Precursors	Differentiation to Microglia-like Cells	
Protocol 1 Muffat et al.	Day − 1 – 0 DMEM/F12 + FBS (15%) +KOSR (5%) +FGF2 (4 ng/ml)	Day 0–14 Neurobasal Medium + N2 (100x) + B27 (50x) + CSF1 (10 ng/ml) + IL-34 (10 ng/ml)					Day 14–44 Neurobasal Medium + N2 (100x) + B27 (50x) + CSF1 (10 ng/ml) + IL-34 (10 ng/ml)	Day 44–74 Neurobasal Medium + N2 (100x) + B27 (50x) + CSF1 (5 ng/ml) + IL-34 (100 ng/ml)	
Protocol 2 Pandya et al.	Day − 2 – 0 mTeSR1	Day 0–4 STEMdiff APEL + VEGF (30 ng/ml) + BMP4 (30 ng/ml) + SCF (40 ng/ml) + Activin A (50 ng/ml)		Day 4–15 STEMdiff APEL + BMP4 (25 ng/ml) + SCF (300 ng/ml) + IL-3 (10 ng/ml) + IL-6 (10 ng/ml) + Flt3L (300 ng/ml) + CSF3 (50 ng/ml)				Day 15–29 (Astrocyte-MGL coculture) IMDM + FBS (10%) + IL-3 (20 ng/ml) + CSF1 (20 ng/ml) + CSF2 (20 ng/ml)	
Protocol 3 Abud et al.	Day − 1 – 0 TeSR-E8	Day 0–2 IMDM/F12 (1:1) + ITSG-X (2%) + FGF2 (50 ng/ml) + BMP4 (50 ng/ml) + Activin A (12.5 ng/ml) + RhoKI (1 μM) + LiCl (2 mM)	Day 2–4 IMDM/F12 (1:1) + ITSG-X (2%) + FGF2 (50 ng/ml) + VEGF (50 ng/ml)	Day 4–10 IMDM/F12 (1:1) + ITSG-X (2%) + FGF2 (50 ng/ml) + VEGF (50 ng/ml) + TPO (50 ng/ml) + SCF (10 ng/ml) + IL-3 (10 ng/ml) + IL-6 (50 ng/ml)			Day 10–14 DMEM/F12 (1:1) + ITSG (2%) + insulin (5 μg/ml) + N2 (0.5%l) + B27 (2%) + CSF1 (25 ng/ml) + IL-34 (100 ng/ml) + TGF-β (50 ng/ml)	Day 14–35 DMEM/F12 (1:1) + ITSG (2%) + insulin (5 μg/ml) + N2 (0.5%l) + B27 (2%) + CSF1 (25 ng/ml) + IL-34 (100 ng/ml) + TGF-β (50 ng/ml)	Day 35–38 DMEM/F12 (1:1) + ITSG (2%) + insulin (5 μg/ml) + N2 (0.5%l) + B27 (2%) + CSF1 (25 ng/ml) + IL-34 (100 ng/ml) + TGF-β (50 ng/ml) + CD200 (100 ng/ml) + CXCL1 (100 ng/ml)
Protocol 4 Douvaras et al.	Day − 3 – 0 mTeSR1	Day 0–4 mTeSR1 + BMP4 (80 ng/ml)		Day 4–6 StemPro-34 SFM + FGF2 (25 ng/ml) + VEGF (80 ng/ml) + SCF (100 ng/ml)	Day 6–14 StemPro-34 SFM + SCF (50 ng/ml) + TPO (5 ng/ml) + Flt3L (50 ng/ml) + IL-3 (50 ng/ml) + CSF1 (50 ng/ml)		Day 14–25/50 StemPro-34 SFM + Flt3L (50 ng/ml) + CSF1 (50 ng/ml) + CSF2 (25 ng/ml)	Day 25/50–45/65 RPMI-1640 + CSF2 (10 ng/ml) + IL-34 (100 ng/ml)	
Protocol 5 Haenseler et al.	Day − 2 – 0 mTeSR1	Day 0–4 mTeSR1 + VEGF (50 ng/ml) + BMP4 (50 ng/ml) + SCF (20 ng/ml)		Day 4–14 X-VIVO15 + IL-3 (25 ng/ml) + CSF1 (100 ng/ml)			Day 14–28/56 X-VIVO15 + IL-3 (25 ng/ml) + CSF1 (100 ng/ml)	Day 28/56–42/70 Advanced DMEM/F12 + N2 (1%) + IL-34 (100 ng/ml) + CSF1 (10 ng/ml)	
Protocol 6 Takata et al.	Day − 1 – 0 mTeSR1	Day 0–2 StemPro-34 SFM + Transferrin (200 mg/ml) + BMP4 (5 ng/ml) + VEGF (50 ng/ml) + CHIR (2 μM)	Day 2–4 StemPro-34 SFM + Transferrin (200 mg/ml) + BMP4 (5 ng/ml) + VEGF (50 ng/ml) + FGF2 (20 ng/ml)	Day 4–6 StemPro-34 SFM + Transferrin (200 mg/ml) + VEGF (15 ng/ml) + FGF2 (5 ng/ml)	Day 6–12 StemPro-34 SFM + Transferrin (200 mg/ml) + VEGF (10 ng/ml) + FGF2 (10 ng/ml) + SCF (50 ng/ml) + IL-3 (20 ng/ml) + IL-6 (10 ng/ml) + DKK1 (30 ng/ml)	Day 12–16 StemPro-34 SFM + Transferrin (200 mg/ml) + FGF2 (10 ng/ml) + SCF (50 ng/ml) + IL-3 (20 ng/ml) + IL-6 (10 ng/ml)	Day 16–25 IMDM/F12 (3:1) + N2 (1%) + B27 (2%) + CSF1 (50 ng/ml)	Day 25–46 (Neuron-MGL coculture) not reported

**Table 2:** Detailed summary of the molecular markers and functional tests used to characterize microglia-like cells

	Hematopoietic Progenitors	Microglia Precursors	Microglia-like Cells	Efficiency	Reproducibility
Protocol 1 – Muffat et al.	Morphology: cystic structure ICC: CD144 (*CDH5*), CD117 (*KIT*), CD41 (*ITGA2B*), CD235a (*GYPA*), PU.1 (*SPI1*)	Morphology: round, vacuolated cells, compact nucleus, filopodia, membrane ruffles ICC: CD11b (*ITGAM*), IBA1 (*AIF1*), PU.1 Flow: CD11b, IBA1, CD45 (*PTPRC*) Phagocytosis: latex bead assay Proliferation: EdU assay	Morphology: multiple thin first-order branches terminated by membrane ruffles ICC: TMEM119, P2Y12 (*P2RY12*), IBA1, CD45 Cytokines (CTRL, LPS + IFN-γ): Cytokine Array R&D Transcriptional Profile: RNA-Seq	Yield: 0.5-4x Purity: 97%	15 hiPSC-lines 3 hESC lines
Protocol 2 - Pandya et al.	Morphology: floating cells ICC: CD34, CD43 (*SPN*), CD45	not reported	Flow: CD11b, IBA1, CD45, CX3CR1, HLA-DR; Negative: CD80, CD86, CD206, CD200R Cytokines (CTRL, LPS): TNF-α, kit unspecified Phagocytosis: pHrodo-*E.coli* ROS: CellROX assay Transcriptional Profile: Microarray	Yield: 0.8-3x Purity: 9% (in mixed glial culture)	2 hiPSC lines
Protocol 3 – Abud et al.	Morphology: endothelial cells Flow: CD41 (*ITGA2B*), CD43, CD235a	ICC: PU.1, TREM2 Flow: CD11b (9%), CD45 (65%), CX3CR1 (22%); negative: CD117	Morphology: high nucleus to cytoplasm ratio ICC: PU.1, CX3CR1, TREM2, P2Y12, TGFBR1, PROS1, MERTK, ITGB5 Flow: CD11b^int^, CD45^low-int^ Phagocytosis: pHrodo-*E.coli*, pHrodo-hS (CD11b-dependent, MERTK-independent) Cytokines (CTRL, LPS, IL-1β, IFN-γ): V-PLEX cytokine 30-plex Calcium Signaling: ADP-response (P2Y12-negated) Transcriptional Profile: RNA-Seq Transplantation: into MITRG mice	Yield: 30-40x Purity: 97%	10 hiPSC lines
Protocol 4 - Douvaras et al.	Morphology: adherent endothelial cells Flow: CD309 (*KDR*), CD235a (up to 6%)	Morphology: floating cells Flow: CD14, CD45, CX3CR1 (70%)	Morphology: ramified cells with motile processes ICC: CD11c, IBA1, P2Y12, TMEM119 Flow: CD11b, CD11c, CX3CR1, P2Y12 Phagocytosis: latex beads Cytokines (CTRL): Human XL Cytokine Array Kit Calcium Signaling: ADP-response Transcriptional Profile: RNA-Seq	Yield: 2x Purity: 68% (CD14^+^)	14 hiPSC lines 2 hESC lines
Protocol 5 - Haenseler et al.	Flow: CD34	Morphology: large, vesicle-rich, floating cells Flow: CD14, CD16, CD45, CD86, CD163, HLA-DR Phagocytosis: pHrodo-zymosan	Morphology: ramified (secondary branches) Flow: CD11b, CD11c, CD14, CD45, (CD33), (MERTK); Negative: HLA-DR Cytokines (CTRL, LPS/IFN-γ): Human XL Cytokine Array Kit (R&D), Luminex 100 Bio-Plex System (BioRad)	Yield: 10-43x Purity: ~ 100% (CD14^+^)	4 hiPSC lines
Protocol 6 - Takata et al.	not reported	Morphology: floating, round, vacuolated cells Flow: CD11b, CD14, CD45, CD163, CX3CR1 CyTOF: CD11b, CD14, CD45, CD48, CD62L, CD64, CD115, CD163, CX3CR1, HLA-DR, MARCO, MERTK Phagocytosis: latex beads, Aβ-TAMRA	Morphology: ramified cells ICC: IBA1 FLOW: CX3CR1 (increased expression compared to d25) Phagocytosis: latex beads, Aβ-TAMRA Cytokines (CTRL, LPS): Human Magnetic Luminex Assay (R&D) Transcriptional Profile: none	Yield: n.a. Purity: 94% (CD14^+^/CD45^+^)	1 hiPSC line

### Translational applications of hPSC-derived microglia-like cells

Four of the six basic MGL protocols or derivatives thereof have been applied by several independent research groups for translational studies (summarized in Table 3).

**Table 3:** Summary of publications and preprints using the six basic protocols for the generation of microglia-like cells from human pluripotent stem cells for basic microglia research or translational applications

First author / Year	MGL protocol	mutant hPSCs	main findings
Muffat et al. 2016 [21]	Muffat-MGL	*MECP2*^*−/−*^	smaller microglia diameter.
Gosselin et al. 2017 [33]	Muffat-MGL	n/a	The transcriptome of MGLs closely resembles primary microglia cultured in vitro but differ significantly from microglia ex vivo.
Muffat et al. 2018 [34]	Muffat-MGL	n/a	Infected primitive yolk sac macrophages may act as ZIKV reservoir during pregnancy and establish brain infection upon colonising the developing CNS.
Lin et al. 2018 [35]	Muffat-MGL	*APOE* (ε3 vs. ε4)	*APOE4* perturbs several microglial functions preventing microglia from clearing Aβ from AD brain organoids effectively.
Brownjohn et al. 2018 [36]	Haenseler-MGL	*TREM2*^*T66M*^; ^*−W50C*^	loss of TREM2 surface expression; reduced mature isoforms; reduced TREM2 proteolysis.
Garcia-Reitboeck et al. 2018 [37]	Haenseler-MGL	*TREM2*^*T66M*^; ^*−W50C*^	reduced or absent soluble TREM2 in supernatants.
Xiang et al. 2018 [13]	Haenseler-MGL (modified)	*TREM2*^*R47H*^	normal TREM2 mRNA and protein expression and splicing patterns.
Piers et al. 2019 [38]	Haenseler-MGL (Xiang)	*TREM2*^*T66M*^*;* ^*−W50C*^*;* ^*−R47H*^	metabolic deficits: a reduced mitochondrial respiratory capacity and an inability to perform a glycolytic immunometabolic switch, due to dysregulated PPARγ/p38MAPK signalling.
Hasselmann et al. 2019 [39]	Abud-MGL (McQuade)	*TREM2*^*R47H*^	HPSC-derived hematopoietic progenitors engraft in the postnatal mouse brain and adopt the transcriptional identity of human primary microglia*.* TREM2 mutant microglia displayed reduced migration towards Aβ plaques.
Claes et al. 2019 [40]	Douvaras-MGL (modified)	*TREM2*^*R47H*^*;* ^*+/−*^*;* ^*−/−*^	﻿Isogenic hetero- and homozygous TREM2 knockouts resulted in reduced phagocytosis of *E. coli* fragments in vitro and amyloid plaques in AD mouse brain slices. TREM2^R47H^ mutant MGLs displayed normal phagocytosis.
Mancuso et al. 2019 [41]	Douvaras-MGL (modified)	n/a	HESC-derived monocytes engraft in the postnatal mouse brain and adopt the transcriptional identity of human primary microglia.
Takata et al. 2017 [30]	Takata-MGL	*MEFV*^*M694V*^	increased secretion of pro-inflammatory cytokines; increased ASC-speck formation.

In their original study, Muffat et al. used their novel MGL protocol to investigate the effect of a Rett-syndrome causing *MECP2* mutation on morphological features of MGLs, as previous research had demonstrated that transplanted wild-type bone marrow-derived phagocytes could rescue certain features of the disease [21]. *MECP2*-mutant MGLs were significantly smaller compared to isogenic controls. Further functional analyses were not performed. The same group published a follow-up study, in which they utilized the same MGL protocol to investigate the cell tropism of the Zika virus (ZIKV) [34]. Maternal ZIKV infection during pregnancy is recognized as the cause of epidemic microcephaly and other neurological anomalies in human fetuses. It remains unclear how the ZIKV accesses the highly vulnerable population of neural progenitors of the developing CNS, and which cell types of the CNS represent viral reservoirs. Muffat et al. modeled the interaction of the ZIKV with cells of the fetal CNS by infecting isogenic neural progenitor cells (NPCs), neurons, astrocytes, and MGLs. Interestingly, transduction with the ZIKV elicited cytotoxicity only in NPCs and not in any of the mature cell types. Infected glial cells propagated the ZIKV and maintained the ZIKV load over time, leading to a viral spread to susceptible cells. Moreover, in NPCs and glia, virus RNA was rapidly amplified in the first few days after infection. In contrast, infection with the Dengue virus did not lead to cell death or virus propagation in any cell type, consistent with the fact that maternal dengue virus infection does not elicit teratogenicity. Finally, a coculture of ZIKV infected MGLs with uninfected neural spheroids led to neural spheroid infection. The authors proposed a maternal ZIKV infection model, in which primitive yolk sac macrophages that originate close to the maternal vasculature represent the entry route of the ZIKV in the embryo. Infected primitive macrophages act as a viral reservoir and establish the infection of the fetal brain upon colonizing the developing CNS [34].

Muffat-MGLs were employed by another group to investigate the molecular phenotypes and functional consequences of different Apolipoprotein E (ApoE) isoforms in hPSC-derived neurons, astrocytes, and microglia [35]. ApoE is an essential cholesterol carrier that is involved in lipid transport and injury repair in the brain. ApoE is encoded by the *APOE*-gene. Several polymorphic *APOE* alleles have been described and represent the main genetic determinants of Alzheimer’s disease (AD): individuals carrying the ε4 allele are at increased risk of developing AD compared with those carrying the more common ε3 allele, whereas the ε2 allele decreases the risk. Using CRISPR/Cas9-mediated gene-editing, the authors converted wild-type *APOE3* hiPSCs into isogenic *APOE4* hiPSCs and vice versa. HiPSC-derived neurons, astrocytes, and MGLs exhibited genotype-dependent differential gene expression patterns. Specifically, *APOE4* MGLs were characterized by an upregulation of genes connected with immune responses and a downregulation of genes associated with cell movement and development. Moreover, *APOE4* MGLs developed less and shorter cellular processes and took longer to phagocyte Aβ compared to *APOE3* MGLs. Upon integrating MGLs within brain organoids derived from APP-mutant hiPSCs displaying overt amyloid pathology, *APOE4* MGLs developed longer processes. Since the shorter process length of microglia in AD brains positively correlates with microglial Aβ uptake, these data suggest that *APOE4* MGLs may be less able to sense and respond to extracellular Aβ. In this context, the authors reported significantly lower total amyloid levels in organoids containing *APOE3* MGLs compared to both *APOE4* MGL-containing organoids and organoids without cocultured MGLs. Overall, the data suggest that *APOE4* negatively impacts several aspects of microglial function, hinders microglia from clearing extracellular Aβ from AD brains, and may also affect the brain inflammatory profile [35].

The triggering receptor expressed on myeloid cells 2 (TREM2) is a cell surface receptor of the immunoglobulin superfamily that among neural cell types is differentially expressed on microglia [11]. Genetic studies have identified loss-of-function mutations of TREM2 as the cause for a rare form of early-onset dementia with autosomal recessive inheritance, known as Nasu-Hakola disease [42], and many additional variants as rare but strong risk factors for developing Alzheimer’s disease [43]. Several independent studies have investigated the functional effects of different TREM2 variants in hPSC-derived MGLs [13, 36, 37]. The first four TREM2-related studies are based on the Haenseler MGL protocol, with slight modifications. MGLs generated from patient-derived homozygous and heterozygous TREM2^T66M^ and homozygous TREM2^W50C^ mutant hiPSCs exhibited several genotype-dependent phenotypes [36, 37]: TREM2 immunocytochemistry demonstrated physiological membrane-bound TREM2 expression in wild-type and heterozygous TREM2^T66M^ MGLs but not in homozygous TREM2^T66M^ and TREM2^W50C^ MGLs, indicating a loss of functional receptor surface expression in both types of homozygous mutants. Western blot analysis of whole-cell lysates revealed a genotype-dependent reduction in mature forms and concomitant increase in immature forms of full-length TREM2, thus indicating mutation-dependent effects on protein maturation. Furthermore, western blot analysis of whole-cell lysates using an antibody directed against the carboxy-terminus of TREM2 revealed a genotype-dependent reduction in the generation of the C-terminal fragment of TREM2 following treatment with a γ-secretase inhibitor, marking reduced proteolysis of TREM2, most likely due to reduced cell surface expression and shedding of soluble TREM2. Membrane TREM2 undergoes regulated intramembrane proteolysis (RIP) by the metalloproteases ADAM10 and ADAM17, which releases a soluble TREM2 fragment (see below) and leaves a membrane-bound C-terminal fragment that is a substrate for the γ-secretase complex [36]. In line with this observation, a follow-up study using the same mutant hiPSC-lines confirmed that levels of the soluble TREM2 fragment (the other RIP product) in supernatants of heterozygous TREM2^T66M^ MGLs were reduced compared to wild-type MGLs, and soluble TREM2 was not detected in homozygous TREM2^T66M^ and TREM2^W50C^ MGLs [37]. In another study, Xiang et al. generated MGLs from hiPSCs harboring the heterozygous TREM2^R47H^ variant to test a proposed mechanism of action of this AD risk variant [13]. Results from Trem2^R47H^ knock-in mouse models had demonstrated Trem2 haploinsufficiency with reduced mRNA and protein expression due to the atypical splicing of mouse Trem2^R47H^, which introduces a premature stop codon. Interestingly, TREM2 mRNA and protein expression levels and splicing patterns were normal in mutant hiPSC-derived MGLs and patient-derived brain tissue, indicating that aberrant splicing is a species-specific mechanism that does not translate to human biology, thus highlighting the need for human microglia models [13]. Another Haenseler MGL protocol-based study using different TREM2 mutant hPSCs (TREM2^T66M^;^-W50C^;^-R47H^) confirmed metabolic deficits in mutant MGLs [38]. During homeostasis, microglia mainly rely on oxidative phosphorylation for their energy supply but undergo a metabolic switch to glycolysis upon activation to enable more rapid plasticity to external stimuli [44]. Piers et al. demonstrated that all analyzed TREM2 mutants exhibit altered metabolic functions, including a reduced mitochondrial respiratory capacity and an inability to perform the immunometabolic switch towards increased glycolysis. Moreover, the authors determined that dysregulated PPARγ/p38MAPK signaling underlies the observed phenotypic deficits, specifically in TREM2^R47H^ mutants. The genotype-dependent phenotype was rescued by pharmacological activation of PPARγ using pioglitazone [38]. Claes et al. generated isogenic hetero- and homozygous TREM2 knock-out hPSCs and heterozygous TREM2^R47H^ hPSCs from wild-type hPSCs by CRISPR/Cas9-mediated genome engineering and differentiated them into MGLs according to a modified Douvaras MGL protocol [40]. Both hetero- and homozygous TREM2 knock-out MGLs exhibited a reduced capacity to phagocyte *E. coli* fragments in vitro and amyloid plaques in cocultures with brain cryosections derived from a mouse model of Alzheimer’s disease. In contrast, the phagocytotic abilities of TREM2^R47H^ mutants were not impaired [40]. Lastly, Hasselmann et al. reported that TREM2^R47H^ hPSC-derived hematopoietic progenitors that were derived according to the Abud/McQuade protocols and transplanted into immunodeficient, humanized mice (see above) with features of Alzheimer’s disease (5XfAD) differentiated into bona fide microglia in vivo. After 9 months, transplanted mutant microglia clustered less frequently around Aβ plaques compared to wild-type microglia, although no difference in overall plaque burden was apparent between study groups.

Finally, Takata-MGLs were utilized in the original study to investigate the effect of a familial Mediterranean fever (FMF), causing *MEFV-*mutation on morphological features and functions of MGLs [30]. Cytokine profiling revealed that following LPS stimulation, homozygous *MEFV*-mutant MGL precursors produced significantly higher levels of proinflammatory cytokines, including IL-1β, IL-18, TNFα, and CCL4, compared to heterozygous control MGLs. Moreover, ASC-speck formation, a critical component of the inflammasome, was significantly enhanced in mutant MGL precursors [30].

### Microglia-like cells in the mirror of embryonic development

The use of classical differentiation protocols for deriving MGLs from hPSCs has the great advantage of guiding epiblast-like pluripotent stem cells along precise developmental trajectories into the desired mature cellular phenotype in vitro. Some protocols for the derivation of MGLs were designed to recapitulate the development of primitive hematopoiesis and microglia ontogeny in vitro, and provide preliminary, albeit not unequivocal evidence that the differentiating cells pass through key developmental steps of primitive yolk sac hematopoiesis [21, 23, 28, 29]. In contrast, other researchers initially generated hPSC-derived monocytes, which subsequently adopted microglia-like properties upon exposure to extracellular cues mimicking the CNS environment [25, 40], a method that had been demonstrated previously with primary peripheral blood-derived monocytes [45, 46]. Interestingly, transcriptome analysis revealed that hPSC-derived monocytes [25, 47] resemble transdifferentiated MGLs and human primary microglia rather than monocytes derived from human peripheral blood [40]. Overall, these findings indicate that current protocols for the differentiation of hPSCs into monocytes in vitro may preferentially rely on MYB-independent hematopoiesis, which would favor the production of cells that are part of the primitive over definitive hematopoietic lineages, and give merit to the use of hPSC-derived monocyte-like cells as developmental intermediate when generating microglia. From an embryonic perspective, the use of monocytes remains contentious, at least for some applications, because of the distinct ontogeny of microglia and peripheral tissue macrophages [33].

The onset of gastrulation in humans is marked by the occurrence of the primitive streak (PS) at the posterior end of the bilaminar embryonic disk made up of the epi- and hypoblast. Cells of the epiblast migrate towards the PS, where they ingress into the interior of the embryo to form definitive endoderm and all different types of mesoderm. Mesoderm specification is initiated and driven by dynamic morphogen gradients involving BMP, NODAL/ACTIVIN, FGF2, and WNT signaling along the anterior-posterior axis of the embryo and PS [48]. The epiblast cells, ingressing through the most posterior tip of the early PS, where they are exposed to high levels of BMP4, are specified into extra-embryonic mesoderm from which the hematopoietic program is subsequently initiated. Once induced, extra-embryonic mesodermal cells migrate anteriorly, where they are exposed to FGF2 and WNT inhibitors secreted throughout the anterior-posterior axis and to primitive node-derived NODAL/ACTIVIN, which is located at the anterior end of the PS [48, 49]. While some protocols include transient BMP4, FGF2, and activin A, signaling activation alongside WNT-inhibition to promote patterning of the epiblast cells into early posterior PS cells and extra-embryonic mesoderm [22, 23, 30], no direct verification of this step, such as CDX2 expression, is provided in any of the available MGL protocols. Extra-embryonic mesodermal cells start expressing endothelial cell markers such as KDR and CDH5 and commit towards the related lineages of endothelial and hematopoietic cells [50, 51]. Recent hPSC studies proposed that KDR^+^ mesodermal cells may be dichotomized according to their CD235a (*GYPA*) expression status. While KDR^+^/CD235a^+^ hemogenic endothelial cells subsequently give rise to either primitive CD34^+^/CD43^+^ or transient definitive CD34^+^/CD43^−^ hematopoietic progenitors, KDR^+^/CD235a^−^ hemogenic endothelial cells are committed towards definitive CD34^+^/CD43^−^ hematopoietic progenitors from which, eventually, self-renewing hematopoietic stem cells arise [52, 53]. This fate decision between the two different types of KDR mesoderm is partly mediated by activin A and WNT-signaling according to the migration of mesodermal cells relative to the embryonic signaling centers [52, 53]. Hemogenic progenitors migrate into the blood islands within the extraembryonic yolk sac. From here, primitive erythroid precursors responsible for efficient oxygen delivery to the quickly expanding embryo as well as macrophage and megakaryocyte progenitors emerge during the earliest wave of hematopoiesis [54–56]. Primitive macrophage progenitors delaminate from the hemogenic endothelial cells in the blood islands in a RUNX1-dependent process [55, 57, 58] and critically depend on the expression of the TF PU.1 in conjunction with RUNX, CEBP, and IRF TFs [59–62], while being independent of MYB expression [28, 63]. Among others, primitive macrophage progenitors are characterized by the expression of the surface markers CD34 and CD43. Muffat et al. demonstrate the formation of an EB-fraction containing hemogenic endothelial cells expressing the TF PU.1 alongside the endothelial markers CDH5, KIT, CD41, and CD235a. KDR expression was not reported, nor the offspring of CD34 or CD43 expressing hematopoietic progenitors [21]. Pandya et al. described the emergence of a fraction of CD34^+^/CD43^+^ hematopoietic progenitors that were enriched by FACS for subsequent differentiation steps, while potential prior hemogenic epithelial cells were not characterized [22]. Abud et al. generate CD41^+^/CD43^+^/CD235a^+^ hematopoietic progenitors with a relatively high degree of purity that is further enhanced by FACS for subsequent steps of differentiation [23]. In the protocol by Haenseler et al., common CD34^+^ progenitors emerge that are dependent on the expression of PU.1 and RUNX1 but not MYB, as demonstrated by careful hPSC knock-out studies [28]. The developmental potency of intermediate hemogenic endothelial cells or hematopoietic progenitors to give rise to alternative cell types, including erythrocytes, megakaryocytes, or lymphocytes, was not investigated in any studies related to the generation of MGLs. Upon maturation into primitive macrophages within the yolk sac, cells start upregulating expression of CSF1R and common myeloid cell markers such as CD11b, CD14, and CD45 [64]. Primitive hematopoiesis and subsequent yolk sac maturation of primitive macrophages are dependent on the growth factors SCF, IL-3, IL-6, and CSF-1. Once the circulatory system is established, primitive yolk sac macrophages spread via the bloodstream to populate the developing CNS and other tissues of the embryo, thus giving rise to the fetal tissue-resident macrophages [1–3, 55, 56, 65]. Primitive yolk sac macrophages that have entered the CNS are exposed to niche-specific environmental cues including CSF-1, IL-34, TGF-1β, and CCL2, leading to further maturation into microglia [66].

### Microglia identity in vitro and in vivo

The unique microglia identity is currently best defined as the result of a complex interplay of developmental origin-dependent transcriptional networks and CNS-derived environmental factors [33]. The microglia phenotype in vivo is highly plastic and reactive to environmental changes. According to a simplistic scheme, microglia may be grossly categorized as either homeostatic, differentially activated, or disease-associated. Interestingly, primary microglia cultured in vitro rapidly change their morphology and functions following their isolation from brain tissue, in a manner concomitant with profound transcriptomic changes, including loss of key “signature microglia genes” [33, 67, 68]. Microglia identity is further complicated: bone marrow-derived blood monocytes that cross the blood-brain barrier and enter the brain parenchyma, particularly under certain pathological conditions, may adopt a microglia-like phenotype. All markers proposed to date to differentiate monocyte-derived microglia-like cells from microglia lack the required level of specificity. Due to the inherent challenges in defining a clear-cut microglia identity, the “MGL products” of available hPSC-based differentiation protocols are difficult to rate. Ultimately, microglia cultured in vitro need to be carefully characterized concerning their morphology, marker protein expression, gene expression profile, metabolism, physiological signaling pathways, and functions such as phagocytosis and cytokine secretion – independent of their origin (i.e., brain-tissue, peripheral blood monocytes, or hPSCs). All features must be analyzed in the context of the mono- or coculture system applied and benchmarked against primary cells ex vivo and under identical in vitro culture conditions. Generally, microglia show enhanced activation in vitro, and their response to various stimuli or treatments may differ in vivo. In vitro studies of human microglia represent a powerful tool and are superior over murine microglia with regard to the many species-specific differences (e.g., genetic risk factors for Alzheimer’s disease and other neurodegenerative diseases). Nevertheless, any in vitro derived microglia have to be regarded as “microglia-like”, and translational observations need to be confirmed in more complex in vitro coculture systems or in vivo chimeric humanized mouse models. Importantly, most recent studies have demonstrated that the loss or lack of microglia signature gene expression and the more activated phenotype of microglia in vitro is fully reversible upon transplantation into the mouse brain, irrespective of the in vitro origin of the microglia-like cell. For instance, in vitro cultured primary mouse myeloid cells, with diverse ontogenic origins, extensively engraft the CNS of microglia-deficient mice 2 weeks after transplantation, but only myeloid cells of yolk sac origin fully attain microglial identity upon exposure to the CNS environment [68]. Likewise, hPSC-derived hematopoietic progenitors and MGLs derived according to the Abud/McQuade protocols [23, 24] and monocytes derived from hPSCs according to the Yanagimachi/Douvaras protocols [25, 47] exhibited robust engraftment following transplantation into early postnatal brains of immunodeficient, humanized mice [23, 24, 39, 41]. Engrafted cells adopted a typical microglia morphology and had a whole-genome expression profile that was indistinguishable from primary microglia ex vivo. A recent study reported the innate development of hPSC-derived mesoderm microglia within brain organoids, closely mimicking the transcriptome and response of adult microglia isolated from postmortem human brain tissue [69]. Taken together, hPSC-derived microglia models, ranging from reductionist in vitro studies that overcome significant species-specific differences to complex in vivo studies using chimeric mouse models, are powerful research tools.

## Conclusions

Microglia have arrived surprisingly late at the forefront of stem cell research, but finally, the field has been equipped with a rich set of differentiation protocols for the generation of MGLs from hPSCs. Following the seminal publication by Muffat and colleagues in 2016 [21], several independent protocols for generating MGLs from hPSCs have been developed. Great variations are observed with regard to the media compositions applied, steps taken to enrich developmental precursor populations, and identity of the resulting MGL phenotypes. The reported culture durations range from 38 to 74 days, the yields are highly variable and range from 0.5–40x MGLs compared to the number of input-hPSCs, and the cellular homogeneity of the final MGL population ranges from 9% MGLs in undefined mixed glia cultures to almost pure monocultures of MGLs. These numbers need to be interpreted with caution as markers and tools used for assessments differ amongst studies. Some protocols require mechanical manipulation steps, special devices, coculture with other cell types to induce MGL maturation, EB-formation, or non-defined serum-containing media formulations. Inherently, such measures introduce different degrees of variability and thus hamper the widespread application and reproducibility of the protocols. Ultimately, the protocol most suitable for a particular application has to be chosen individually, depending on the resources of the laboratory and the planned downstream applications.

While this first wave of protocols was seminal and opened new avenues for microglia research, their advent also brought many additional questions into the field. Although providing the best alternative to primary microglia, there is no current consensus on unique proteomic, genetic, and functional signatures required to consider hPSC-derived MGLs worthy representatives of their native counterparts. Moreover, hPSC-derived MGLs more closely resemble fetal than adult microglia, which may hinder the study of age-related neurodegeneration. Recent efforts to study age-related neurodegeneration using hPSC-derived MGLs rely on inserting genetic Alzheimer’s disease risk variants into the genome of hiPSCs before differentiating them into MGLs, or coculturing MGLs with brain organoids derived from hiPSCs with APP mutations for extended culture periods, resulting in overt amyloid pathology (Table 3). Alternative future options may involve strategies similar to those previously employed to induce features of age-related degeneration in hiPSC-derived neurons, including application of ROS or mitochondria-related stressors [70], or the induction of broader aspects of aging by expressing progerin, the premature aging-associated variant of the *LMNA*-gene (coding for the nuclear envelope protein lamin A) [71], which may also lead to premature aging in hiPSC-derived MGLs. An alternative approach may include the direct reprogramming (transdifferentiation) of adult somatic cells, e.g., fibroblasts or monocytes, into microglia-like cells with specific transcription factor cocktails, as direct somatic cell reprogramming, in contrast to reprogramming to pluripotency, does not erase age-related cellular features.

Refined differentiation or reprogramming protocols will eventually allow us to produce pure bulk quantities of microglia from hPSCs and will be a major step towards understanding this essential cell type in health and disease. First, while the in vitro phenotype of microglia, regardless of their origin, remains to a certain degree artificial, the ability to produce large numbers of hPSC-derived MGLs and to place them in complex cocultures with other neural cell types and human brain organoids, or to perform xenotransplantation into immunodeficient, humanized mouse models, provides an excellent platform to pinpoint environmental factors that contribute to the differences between in vitro and in vivo phenotypes of microglia. Ideally, these approaches will aid the identification of enhanced chemically defined culture conditions composed of cytokine or small molecule combinations that enable in vitro culturing of faithful microglia. Second, the addition of MGLs to 2D and 3D CNS coculture models permits us to establish microglia with properties that resemble the in vivo phenotype and also improves previous in vitro models of the human brain that were deficient of blood supply and immune cells in the past. Hence, the in vitro coculture of MGLs with other neural cell types or brain organoids holds great potential for both reductionist cross-over studies in 2D and complex disease models in 3D. Third, hPSC-derived microglia help us to understand the molecular and functional consequences of increased numbers of disease-associated genomic variants and the mutations in genes that lack respective mouse orthologues and are preferentially expressed in microglia. This knowledge is crucial for the conception of translational studies and drug screening platforms. Finally, cell transplantation therapy, applying bespoke gene-corrected microglia or otherwise engineered microglia as efficient vectors for delivering neuroprotective or regenerative functions, may be harnessed for treating a variety of neurological diseases in the future.

## Data Availability

Not applicable.

## Abbreviations

AIF1: Allograft inflammatory factor 1
BMP4: Bone morphogenetic protein 4
C1QA: Complement C1q A chain
CCL2: C-C motif chemokine ligand 2
CD: Cluster of differentiation
CDH5: Cadherin 5
CEBD: CCAAT enhancer-binding protein
CNS: Central nervous system
CSF: Colony-stimulating factor
CX3CR1: C-X3-C motif chemokine receptor 1
EB: Embryoid body
ENTPD1: Ectonucleoside triphosphate diphosphohydrolase 1
FACS: Fluorescent-activated cell sorting
FBS: Fetal bovine serum
Flt3LG: Fms related tyrosine kinase 3 ligand
GAS6: Growth arrest-specific 6
GPR34: G protein-coupled receptor 34
GYPA: Glycophorin A
hESC: human embryonic stem cell
hiPSC: human induced pluripotent stem cell
HLA-DR: Major histocompatibility complex, class II, DR
hPSC: human pluripotent stem cell
IL: Interleukin
IRF: Interferon regulatory factor
KDR: Kinase insert domain receptor
KIT: KIT proto-oncogene, receptor tyrosine kinase
LPS: Lipopolysaccharide
MERTK: MER proto-oncogene, tyrosine kinase
MGL: microglia-like cell
mRNA: messenger ribonucleic acid
NPC: Neural precursor cell
P2RY12: Purinergic receptor P2Y12
PDL: Poly-D-lysine
PROS1: Protein S
PS: Primitive streak
PTPRC: Protein tyrosine phosphatase receptor type C
PU.1: Transcription factor PU.1
qPCR: quantitative real-time polymerase chain reaction
RIP: Regulated intramembrane proteolysis
ROS: Reactive oxygen species
RUNX: RUNX family transcription factor
SCF: Stem cell factor
SPI1: Spi-1 proto-oncogene
TC: Tissue culture
TF: Transcription factor
TGF: Transforming growth factor
TMEM119: Transmembrane protein 119
TNF: Tumor necrosis factors
TREM2: Triggering receptor expressed on myeloid cells 2
VEGF-A: Vascular endothelial growth factor A
WNT: Wingless-related integration site
ZIKV: Zika virus
